# A single-center real-world review of 10 kHz high-frequency spinal cord stimulation outcomes for treatment of chronic pain

**DOI:** 10.1016/j.inpm.2024.100402

**Published:** 2024-03-11

**Authors:** Reza Ehsanian, Victor Wu, Radhika Grandhe, Matthew Valeriano, Timothy R. Petersen, W. Evan Rivers, Eugene Koshkin

**Affiliations:** a Division of Pain Medicine, Department of Anesthesiology & Critical Care Medicine, University of New Mexico School of Medicine, Albuquerque, NM, USA; b University of New Mexico, School of Medicine, Albuquerque, NM, USA; c Tennessee Valley Healthcare System, Veterans Administration, Nashville, TN, USA; d Department of Physical Medicine and Rehabilitation, Vanderbilt University Medical Center, Nashville, TN, USA; e Department of Obstetrics & Gynecology, University of New Mexico School of Medicine, Albuquerque, NM, USA; f Office of Graduate Medical Education, University of New Mexico School of Medicine, Albuquerque, NM, USA

**Keywords:** Chronic pain, Spinal-cord stimulation, High-frequency, Pain management, Back pain

## Abstract

**Objective:**

To compare pragmatic real-world 10-kHz high-frequency spinal cord stimulation (HF-SCS) outcomes at a single academic center to the industry-sponsored SENZA-RCT and Stauss et al. study.

**Methods:**

This single-center retrospective study included patients with refractory back or limb pain trialed and/or permanently implanted with the Nevro HF-SCS system from 2016 to 2021. Demographic and outcome data were obtained from the electronic medical record (EMR) and real-world global database maintained by Nevro Corp. Data obtained from the global database were confirmed using the EMR. Main outcome measures included positive responder status (≥50% patient-reported percentage pain reduction (PRPPR)), improvement in function, improvement in sleep, and reduction in pain medication usage. Comparison groups included patient outcomes from the SENZA-RCT and Stauss et al. study.

**Results:**

Patients (N = 147) trialed with HF-SCS were reviewed, with data available for 137. Positive trialed patient responder rate (≥50% PRPPR) was 77% (106/137, 95CI 70–84%) vs. 87% (1393/1607, 95CI 85–89%) Stauss et al. vs. 93% (90/97, 95CI 88–98%) SENZA-RCT HF-SCS. At the last available follow-up, positive implanted patient responder rate was 73% (58/80, 95CI 63–82%) vs. 78% (254/326, 95CI 73–82%) Stauss et al. vs. 79% (71/90, 95CI 70–87%) SENZA-RCT HF-SCS. Sixty-seven percent (59/88, 95CI 57–77%) reported improved function vs. 72% (787/1088, 95CI 70–75%) Stauss et al.; 45% (31/69, 95CI 33–57%) reported improved sleep vs. 68% (693/1020, 95CI 65–71%) Stauss et al. and 16% (9/56, 95CI 6–26%) reported decrease in medication use vs. 32% (342/1070, 95CI 29–35%) Stauss et al.

**Conclusion:**

Patient responder rates in this retrospective pragmatic real-world study of HF-SCS are consistent with previous industry-sponsored studies. However, improvements in quality-of-life measures and reduction in medication usage were not as robust as reported in industry-sponsored studies. The findings of this non-industry-sponsored, independent study of HF-SCS complement those of previously published studies by reporting patient outcomes collected in the absence of industry sponsorship.

## 1 Introduction

Inadequate treatment of chronic pain often leads to diminished function, quality-of-life, and productivity [1,2]. Lower back pain and/or leg pain (LBP-LP) are common causes of chronic pain, representing an increasingly pervasive health issue worldwide [[3], [4], [5]]. Treatment is often multimodal and the escalation of treatment on the Pain Management Continuum Model (PMCM) [6] includes medications, physical therapy, percutaneous interventions, and surgery [7]. Unfortunately, long term pain control remains elusive for many patients [8]. Many patients with chronic and disabling back pain despite extensive conservative management are not surgical candidates; this condition is referred to as nonsurgical refractory back pain (NSRBP) [9]. For patients who undergo surgical intervention for spine conditions, up to 40% have persistent pain or develop new LBP-LP, a phenomenon historically known as failed back surgery syndrome (FBSS) [[10], [11], [12], [13]], now termed persistent spinal pain syndrome (PSPS) [14]. NSRBP and PSPS of the lumbosacral spine are common indications for spinal cord stimulation (SCS) for chronic pain in the United States [15]. SCS is a treatment modality utilized to aid in the management of chronic intractable LBP-LP, with NRSBP and PSPS patients potentially having the greatest benefit from this treatment [16].

For over five decades, SCS has been utilized to disrupt pain signaling in the setting of refractory chronic pain that is not responsive to other treatment modalities [17,18]. Conventional SCS utilizes electrical impulses ranging from 40 to 1200 Hz, termed low-frequency SCS (LF-SCS) [19]. LF-SCS is a cost-effective treatment modality with appreciable pain relief (at least 50% improvement from baseline) for LBP-LP in 30–50% of patients [19]. Significant issues with LF-SCS remain, as a large proportion of patients report limited or no pain relief [[18], [19], [20], [21]]. Additionally some LF-SCS settings produce paresthesias that patients may find uncomfortable, leading to decreased treatment satisfaction [22].

Growing evidence has demonstrated efficacy of novel SCS frequencies [23]. 10-kHz SCS, termed high-frequency SCS (HF-SCS) has been proposed and demonstrated in clinical trials as a safe and effective alternative to LF-SCS [24]. Although the mechanism of HF-SCS is not completely understood, it does not rely on producing paresthesia for pain relief [23,25,26]. Notably, HF-SCS has demonstrated superiority to both medication management and LF-SCS in the treatment of PSPS and NSRBP [27,28].

The Senza™ 10-kHz SCS system designed by Nevro Corporation is an FDA approved HF-SCS device indicated in the treatment of chronic LBP-LP secondary to PSPS or intractable LBP-LP, and in those with NSRBP [16]. The SENZA-RCT demonstrated superiority of HF-SCS to LF-SCS in the management of refractory leg and back pain in both short- and long-term follow-up [28]. A subsequent real-world multi-center pragmatic observational study of HF-SCS patient outcomes [29] (Stauss et al. study) demonstrated effective and sustained pain relief consistent with the findings of the SENZA-RCT.

Although previous studies investigating HF-SCS are scientifically sound, these studies were industry sponsored and may be vulnerable to biases such as exaggeration of positive outcomes and/or withholding of negative data [30,31]. To our knowledge, there has not been a pragmatic study of HF-SCS outcomes without industry funding or sponsorship. This study assesses the efficacy of HF-SCS for the management of chronic refractory back and leg pain as a non-industry sponsored study.

## 2 Methods

### 2.1 Literature review

We conducted a narrative review of existing PubMed indexed HF-SCS literature from January 2015 to February 2023 using the MeSH terms “HF-SCS”, “HF10”, “High-Frequency Spinal Cord Stimulation”, “10-kHz spinal cord stimulation”, and “10,000 Hz spinal cord stimulation” in order to determine whether there were existing non-industry sponsored, pragmatic studies investigating HF-SCS treatment for chronic pain. To determine industry sponsorship, we reviewed the conflicts of interest in individual studies and whether the study or author had direct affiliations or funding with a company or group that manufactured and/or produced HF-SCS devices. We corroborated our manual review with librarians from the University of New Mexico Health Sciences Library and Informatics Center, who conducted their own review of existing HF-SCS literature. We determined that our study is indeed the first non-industry sponsored pragmatic study assessing HF-SCS outcomes for chronic pain.

### 2.2 Study design and setting

This is a single-center, retrospective study analyzing the electronic medical record from the University of New Mexico Hospital (UNMH) (Albuquerque, NM, USA) as well as a real-world database, maintained by Nevro Corporation (Redwood City, CA, USA), populated with records from patients trialed and/or permanently implanted with a Senza™ HF-SCS system. The Human Research Review Committee at the University of New Mexico Health Sciences Center reviewed and approved this study (Study ID 22–162) with waiver of the requirement for informed consent and Health Insurance Portability and Accountability Act authorization. Data from pertinent articles identified from the literature search described in section 2.1 (SENZA-RCT and Stauss et al.) were compared directly to the data from UNMH patients collected and analyzed in this study.

### 2.3 Selection criteria

This is a retrospective analysis of records of consecutive patients from UNMH with back and/or limb pain who were trialed and/or permanently implanted with a Senza™ HF-SCS device between October 2016 and December 2021.

### 2.4 Intervention

The SCS device trial and implantation procedure used in this study is based on recommended clinical standard procedure as described in the SENZA-RCT [28] and The Neurostimulation Appropriateness Consensus Committee Guidelines [32].

### 2.5 Outcome measures

The Nevro database and UNMH electronic medical record were queried for specific patient parameters, including region(s) affected and patient-reported percentage pain reduction (PRPPR) both post-trial and post-permanent implantation (based on percentage scale with 0% as no relief and 100% as complete relief, in 5% increments). Positive trialed responder status and positive permanent implant responder status was determined if patients reported ≥50% PRPPR.

The Nevro database included a phone questionnaire with the following questions:

Improvements in function (measured as yes/no), improvements in sleep (yes/no), changes in medication (increased/decreased/no change), and date of last follow-up.

### 2.6 Follow-up

This study used data from the Nevro database collected through a proactive call cadence similar to those used in previous studies [28,29], which included phone calls from a device expert within the first 6–8 weeks of implantation to optimize therapy. This study also used data collected from medical records documenting clinical care for these patients in outpatient pain management and/or spine health clinic at UNMH.

### 2.7 Data integrity

For purposes of data validation and integrity, 50% of the subjects in the commercial database were randomly selected and then cross referenced with the corresponding UNMH maintained EMR. The cross-referenced data included patient demographic information and review of clinical charts to confirm responder or non-responder status.

### 2.8 Statistical methods

Data from both UNMH records and the real-world database were anonymized, compiled, and analyzed in Microsoft Excel 2016 (Microsoft, Redmond, WA, USA) and GraphPad Prism (Version 9.3.1, GraphPad Software, Boston, MA, USA). We present patient demographic information via routine descriptive statistics, including count and percentage of pain regions. Similarly, we evaluated pain relief and quality-of-life data using absolute counts, percentages, and associated medians and 95% confidence intervals when applicable. When comparing our data to the Stauss et al. study and SENZA-RCT, some parameters such as 95% confidence intervals were not included in the original manuscripts and were subsequently calculated using the data provided in the manuscripts. UNMH PRPPR, quality of life, and medication change comparisons to the Stauss et al. study and the SENZA-RCT were conducted via chi-square analysis and Fisher's exact tests (Table S1).

## 3 Results

### 3.1 Patient cohort

**Fig. 1:**
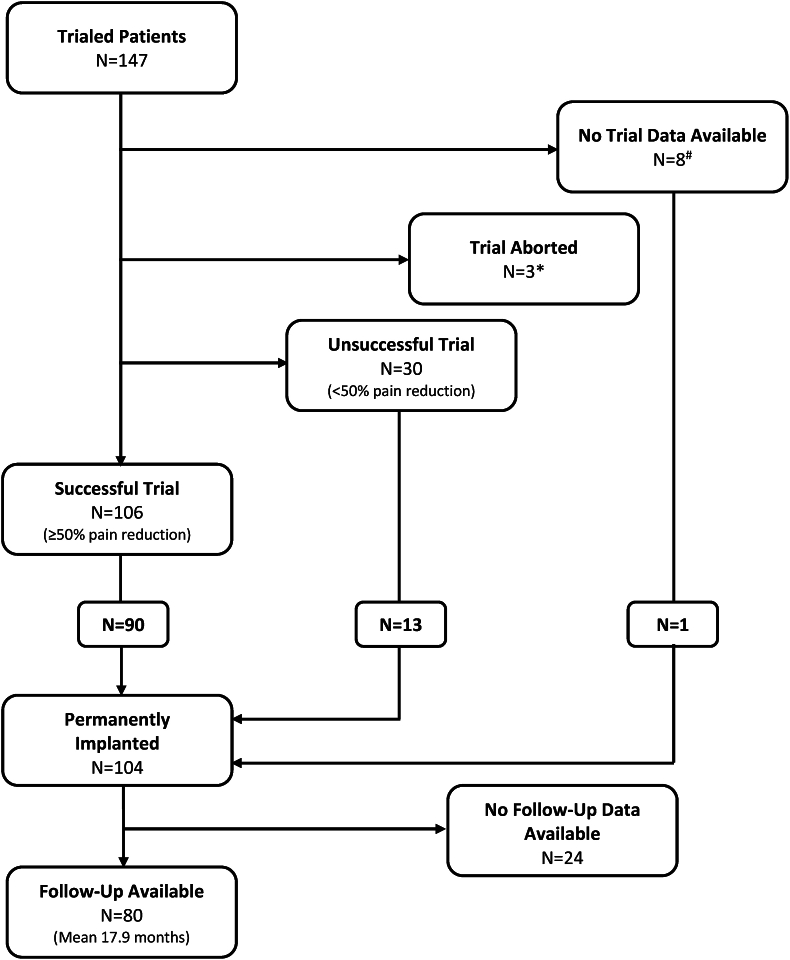
CONSORT Diagram of Patients Trialed and Permanently Implanted Patient data was compiled from the UNMH electronic medical record and real-world database maintained by Nevro Corporation. A successful trial was defined as at least 50% Patient Reported Percentage Pain Reduction. *3 patients who aborted their trial, pain relief data was unavailable for 2 patients. ^#^8 patients had no follow-up data available, resulting in a total of 10 patients trialed who had no reported pain relief outcomes.

We reviewed consecutive records of 147 patients who underwent HF-SCS trials. PRPPR was unavailable for 8 patients (Fig. 1). Three patients aborted their trials before the end of the trial period, with PRPPR unavailable for 2 of these patients (Fig. 1). In total, 10 patients had unavailable PRPPR data, resulting in 137 trialed patients with available pain relief data. Of the 147 patients initially trialed, 106 had a successful trial (≥50% PRPPR) (Fig. 1). One hundred four were permanently implanted with a HF-SCS device including 90 who had undergone successful trial, 13 who had undergone unsuccessful trial, and 1 with unavailable trial data (Fig. 1). A portion of patients who had less than 50% PRPPR continued with permanent implantation. This methodology is consistent with that of the SENZA-RCT, allowing for inclusion of patients with clinical improvement who did not necessarily reach 50% PRPPR. Eighty patients with permanent HF-SCS implants had available PRPPR data (Fig. 1). The mean time between implantation and the last visit was 17.9 months (min 0.5 months, max 62.6 months).

### 3.2 Patient demographics

Patient demographics of trialed (N = 147) and permanently implanted (N = 104) patients at UNMH are presented in columns 1 and 2 of Table 1. Demographic characteristics of the SENZA-RCT HF-SCS permanent implant group (N = 92) are presented in column 3 of Table 1 for comparison. The demographics for trialed patients were not reported in the SENZA-RCT and therefore not available for comparison. Patient demographics were not reported in the Stauss et al. study and therefore not available for comparison. The 147 UNMH patients initially trialed had a mean age of 64 years (SD ± 15), while the 104 permanently implanted patients had a mean age of 65 years (SD ± 14), versus the 92 patients in the SENZA-RCT HF-SCS group who had a mean age of 55 years (SD ± 12) (Table 1). Fifty-two percent of UNMH trialed (77/147) and permanently (54/104) implanted patients were female, while 62% (57/92) of the SENZA-RCT HF-SCS patients were female. Forty-eight percent (71/147) of UNMH trialed patients and 42% (44/104) of UNMH permanently implanted patients had undergone previous back surgery, versus 87% (80/92) of the SENZA-RCT HF-SCS patients (Table 1).

**Table 1:** UNMH vs. SENZA-RCT Patient Demographics.

Groups	UNMH Trialed	UNMH Implanted	SENZA-RCT HF-SCS Implanted
	N = 147	N = 104	N = 92
Age, mean (SD), yrs	64 (15)	65 (14)	55 (12)
Female	77 (52%)	54 (52%)	57 (62%)
Prior Back Surgery	71 (48%)	44 (42%)	80 (87%)

Comparison of UNMH and SENZA-RCT patient demographics including age, sex, and prior back surgery status. UNMH data was collected from real-world database maintained by Nevro Corporation and confirmed with UNMH electronic medical record. Demographics for trialed patients were not reported in the SENZA-RCT. Patient demographics were not reported in the Stauss et al. study. SENZA-RCT HF-SCS Implanted: Patients in SENZA-RCT assigned to receive HF-SCS permanent implant.

### 3.3 Patient characteristics: pain region

Pain region of trialed (N = 147) and implanted patients (N = 104) at UNMH are presented in columns 1 and 2 of Table 2. Pain region of the Stauss et al. study patients (N = 1640) are presented in column 3 of Table 2 for comparison. The manner in which pain region was reported in the SENZA-RCT and the database used in this study differed and therefore could not be compared. Of the 147 trialed UNMH patients, 69% (101/147, 95CI 61–76%) reported combined back and leg pain, 14% (21/147, 95CI 9–20%) reported predominant back pain, and 9% (13/147, 95CI 4–13%) reported predominant leg pain. Eight percent (12/147, 95CI 4–13%) of patients reported other pain regions, including neck, arm, and shoulder pain. Of the 104 implanted UNMH patients, 68% (71/104, 95CI 59–77%) reported combined back and leg pain, 14% (15/104, 95CI 8–21%) reported predominant back pain, and 11% (11/104, 95CI 5–16%) reported predominant leg pain. Seven percent (7/104, 95CI 2–12%) reported other pain regions. Among the Stauss et al. study trialed patients, 43% (713/1640, 95CI 41–46%) had combined back and leg pain, 27% (449/1640, 95CI 25–30%) reported predominant back pain, and 13% (207/1640, 95CI 11–14%) reported predominant leg pain. Seventeen percent (271/1640, 95CI 15–18%) reported other regions of pain. The total number of patients in these categories were calculated based on the percentage reported in the Stauss et al. study and the total of 1640, as the counts were not reported in the study.

**Table 2:** UNMH vs. Stauss et al. Study Patient Characteristics.

Groups	UNMH Trialed	UNMH Implanted	Stauss et al. Study Trialed
**Pain Region**	**N = 147**	**N = 104**	**N = 1640**
Combined Back + Leg Pain	101 (69%; CI 61%–76%)	71 (68%; CI 59%–77%)	713 (43%; CI 41%–46%)
Predominant Back	21 (14%; CI 9%–20%)	15 (14%; CI 8%–21%)	449 (27%; CI 25%–30%)
Predominant Leg	13 (9%; CI 4%–13%)	11 (11%; CI 5%–16%)	207 (13%; CI 11%–14%)
Other	12 (8%; CI 4%–13%)	7 (7%; CI 2%–12%)	271 (17%; CI 15%–18%)
**Neuromodulation Experience**	**N = 140**	**N = 100**	**N = 1596**
Prior Experience	24 (17%; CI 11%–23%)	14 (14%; CI 7%–21%)	381 (24%; CI 19%–22%)
No Prior Experience	116 (83%; CI 77%–89%)	86 (86% CI 79%–93%)	1269 (80%; CI 78%–81%)

Comparison of patient characteristics including predominant pain region and prior neuromodulation experience represented as percentage with 95% confidence interval (CI) between UNMH trialed, UNMH implanted, and Stauss et al. study trialed patients. The manner in which pain region was reported in the SENZA-RCT differed from the manner in which the database tracked pain region in the Stauss et al. study and our current study; and therefore could not be compared.

### 3.4 Patient characteristics: neuromodulation experience

Prior neuromodulation experience among trialed (N = 140) and implanted patients (N = 100) at UNMH are presented in columns 1 and 2 of Table 2. Prior neuromodulation experience among Stauss et al. study patients (N = 1596) are presented in column 3 of Table 2 for comparison. Seventeen percent (24/140, 95CI 11–23%) of UNMH trialed patients and 14% (14/100, 95CI 7–21%) of UNMH implanted patients had previous neuromodulation experience, compared to 24% (381/1596, 95CI 19–22%) of the patients in the Stauss et al. study. The Stauss et al. study did not report pain region and neuromodulation experience data for patients with permanent implants, while in the SENZA-RCT, there was no prior neuromodulation experience allowed by inclusion criteria.

### 3.5 Spinal cord stimulator trial outcomes at UNMH

**Fig. 2:**
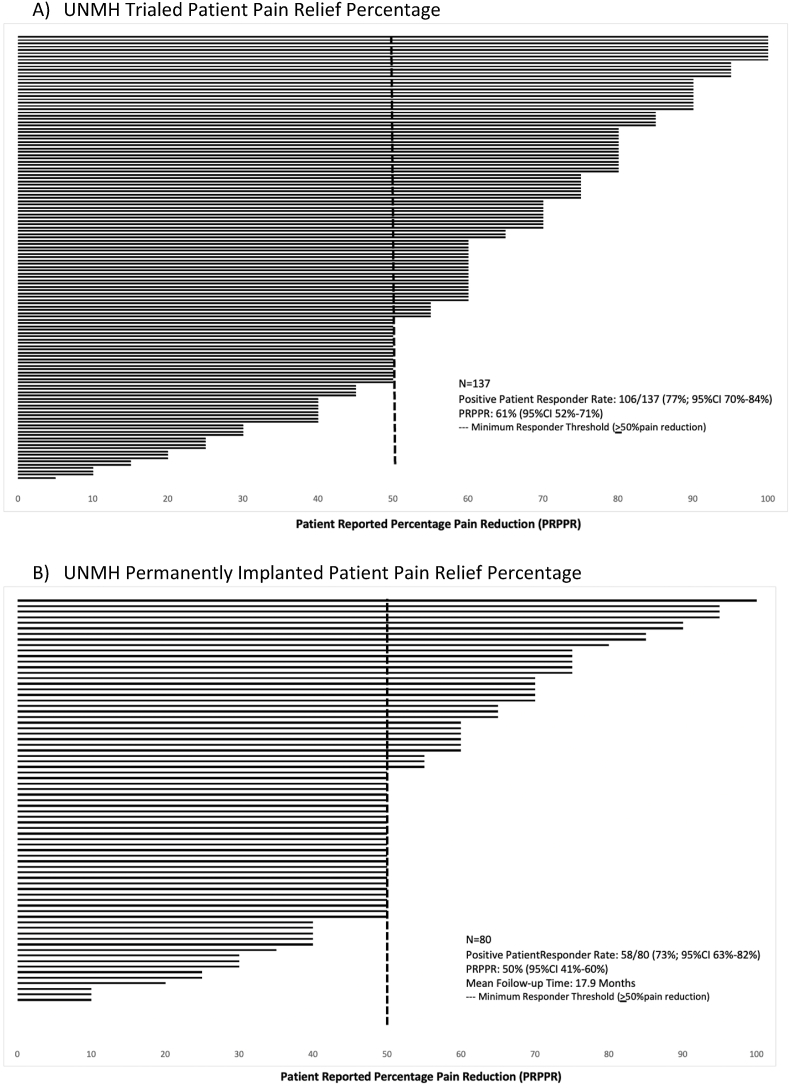
UNMH Patient Responder Rate Bar graphs demonstrating UNMH trialed patient reported percentage pain relief (PRPPR) from baseline (A) and UNMH implanted PRPPR (B). A threshold of 50% PRPPR was used for responder status (dotted line).

At the end of the trial period, outcome data were available for 137 patients. Of the patients with available trial data, 77% (106/137, 95CI 70–84%) of patients reported ≥50% PRPPR (positive patient response), with a mean of 61% PRPPR (95CI 52–71%). (Fig. 2a).

### 3.6 Spinal cord stimulator permanent implant outcomes at UNMH

Data were available for 80 of the permanently implanted patients at last follow-up. Seventy-three percent (58/80, 95CI 63–82%) of patients reported ≥50% PRPPR (positive patient response) following permanent implantation, with a mean of 50% PRPPR (95CI 41–60%). The mean follow-up time was 17.9 months (0.5–62.6 months) (Fig. 2b).

### 3.7 Spinal cord stimulator trial patient responder rate for UNMH versus Stauss et al. study versus SENZA-RCT

**Fig. 3:**
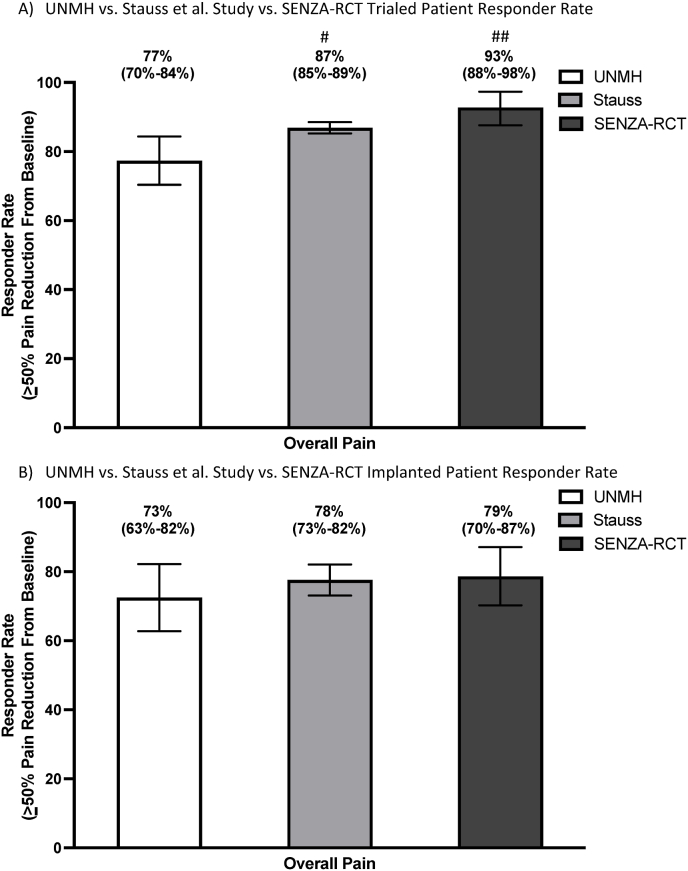
UNMH vs. Stauss et al. Study vs. SENZA-RCT Patient Responder Rate A) UNMH vs. Stauss et al. Study vs. SENZA-RCT Trialed Patient Responder Rate ^#^A statistically significant difference was found when comparing trial outcomes between UNMH vs. Stauss et al. study (p < 0.05) ^##^A statistically significant difference was found when comparing trial outcomes between UNMH vs. SENZA-RCT (p < 0.05) B) UNMH vs. Stauss et al. Study vs. SENZA-RCT Implanted Patient Responder Rate Comparison between UNMH, Stauss et al. Study, and SENZA-RCT HF-SCS group responder rate among all patients permanently implanted with HF-SCS device. Responder rate is represented as percentage with 95% confidence interval (in parentheses). Responder was defined as at least 50% Patient Reported Percentage Pain Reduction. No statistically significant difference was found when comparing permanent implant outcomes between UNMH vs. Stauss et al. study vs. SENZA-RCT (p > 0.05).

The trialed patient responder rate among UNMH patients was compared to those of the Stauss et al. study and SENZA-RCT, using a benchmark of ≥50% pain reduction from baseline as indicative of a positive response (Fig. 3a). Seventy-seven percent (106/137, 95CI 70–84%) of UNMH patients had a positive trial. Eighty-seven percent (1393/1607, 95CI 85–89%) of the Stauss et al. study patients had a positive trial. Ninety-three percent (90/97, 95CI 88–98%) of SENZA-RCT HF-SCS patients had a positive trial. A statistically significant difference was found when comparing trial outcomes between UNMH vs. Stauss et al. study (p < 0.006) and vs. SENZA-RCT (p < 0.002). The calculations for these P-values are shown in Supplemental Fig. 1.

### 3.8 Spinal cord stimulator permanent implant patient responder rate for UNMH versus Stauss et al. study versus SENZA-RCT

The implanted patient responder rate among UNMH patients was compared to those of the Stauss et al. study and SENZA-RCT, using a benchmark of ≥50% pain reduction from baseline as indicative of a positive response (Fig. 3b). Seventy-three percent (58/80, 95CI 63–82%) of UNMH patients were positive responders at last follow-up (mean 17.9 months post implantation). Seventy-eight percent (254/326, 95CI 73–82%) of Stauss et al. study patients, and 79% (71/90, 95CI 70–87%) of SENZA-RCT HF-SCS patients were positive responders at 12 months post implantation. No statistically significant difference was found when comparing permanent implant outcomes between UNMH vs. Stauss et al. study vs. SENZA-RCT (p = 0.54). The calculations for these P-values are shown in Supplemental Fig. 1.

### 3.9 Quality-of-life parameters and medication changes for UNMH versus Stauss et al. study

**Fig. 4:**
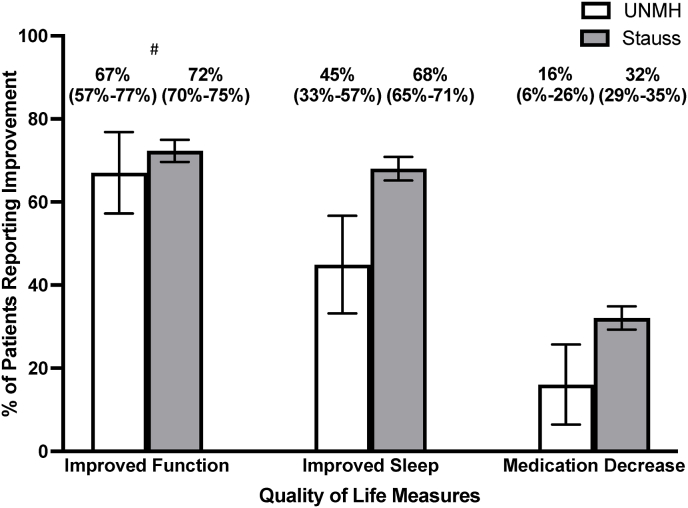
UNMH vs. Stauss et al. Study Patient Quality of Life and Medication Changes Comparison between UNMH and Stauss et al. Study for quality-of-life parameters and medication changes among all patients permanently implanted with HF-SCS device ^#^No statistical difference was found between UNMH vs. Stauss et al. study (p = 0.29) There exists a statistical difference between UNMH vs. Stauss et al. study with regards to improved sleep and medication decrease (p < 0.05).

Quality-of-life parameters and changes in pain medication use among permanently implanted UNMH patients were compared to the Stauss et al. study patients (Fig. 4). Sixty-seven percent (59/88, 95CI 57–77%) of UNMH patients reported functional improvement, 45% (31/69, 95CI 33–57%) reported improved sleep, and 16% (9/56, 95CI 6–26%) reported a decrease in pain medication usage post-implantation (Fig. 4). Seventy-two percent (787/1088, 95CI 70–75%) of Stauss et al. study patients reported functional improvement, 68% (693/1020, 95CI 65–71%) reported improved sleep, and 32% (342/1070, 95CI 29–35%) reported decreased pain medication usage (Fig. 4). Confidence intervals for the parameters from the Stauss et al. study were derived from calculating absolute number of responders based on reported percentages, as the study did not include this data. A statistically significant difference was found for improvement in sleep and decreased medication use with UNMH patients reporting less improvement in sleep and less decrease in pain medication use (p < 0.05). A statistically significant difference did not exist for improved function between the UNMH patients and the patients in the Stauss et al. study (p = 0.29). The calculations for these P-values are shown in Supplemental Fig. 1.

## 4 Discussion

This publication represents the first non-industry sponsored study examining the real-world efficacy of HF-SCS in the treatment of chronic back and leg pain. We conducted a single-center retrospective review of 147 consecutive patients over a five-year period to determine the benefit of HF-SCS for PRPPR at a mean follow-up of approximately 18 months. We compared results from this study to the industry-sponsored Stauss et al. study and the SENZA-RCT [28,29]. These studies were used for comparison not only because they utilized the same HF-SCS device manufacturer, but they are also the most comprehensive investigations assessing HF-SCS treatment for chronic back and leg pain to date. The Stauss et al. study was a retrospective, multicenter review of patients trialed and implanted with a HF-SCS device. The SENZA-RCT was a multicenter, prospective, randomized control trial establishing the efficacy of HF-SCS and its superiority over LF-SCS in the treatment of chronic trunk and limb pain. Both studies used derivations of the visual analog scale (VAS) and numeric rating scale (NRS) to measure pain intensity at baseline, 3-, 6-, and 12-months post-implantation.

The trialed patient characteristics in this investigation were similar to those of the Stauss et al. study, with differences primarily in pain region. There was a larger proportion of patients experiencing combined back and leg pain among the UNMH patients, with a larger proportion of the Stauss et al. patients experiencing predominant back pain and other pain regions.

Notably, our patient demographics consisted of significantly fewer patients who had prior back surgery when compared to the SENZA-RCT. This may be because our study is a real-world study while the SENZA-RCT had more stringent enrollment criteria. It would be interesting to compare the prevalence of patients with prior back surgery in this study with that of the Stauss et al. study. However, this data was not provided and therefore not available for review. Otherwise, the patient demographics of this investigation were comparable to the SENZA-RCT.

Our data show a statistically significant difference in trialed patient responder rate among UNMH patients versus Stauss et al. study (p = 0.006) and SENZA-RCT patients (p = 0.002). Using the same trial period and a ≥50% pain reduction from baseline threshold for positive responder status, the Stauss et al. study and SENZA-RCT showed similar trialed patient responder rates, while our study demonstrates a lower trialed patient responder rate. This observed difference may be due to the fact that the Stauss et al. study and SENZA-RCT calculated pain reduction based on changes in the VAS and NRS after therapy, while UNMH patients were asked to report a PRPPR from baseline after therapy. PRPPR and calculated changes of VAS and NRS are often related, but not fully interchangeable [33,34]. The difference in trialed patient responder rate may also be due to a smaller patient population being sampled in the UNMH study. Our data show a statistically equivalent permanent implant responder rate among UNMH patients versus Stauss et al. study and SENZA-RCT patients when utilizing similar definitions for positive response (p = 0.54).

The current study also examines the effect of HF-SCS on pain medication use changes and quality-of-life parameters including functional and sleep improvement, similar to the Stauss et al. study. The SENZA-RCT reported specifically on changes in opioid medication, but the Stauss et al. study and UNMH patients were surveyed regarding decrease in overall pain medication use. When compared to the outcomes reported by the Stauss et al. study, our results reflect a statistically equivalent degree of functional improvement (p = 0.29), with a statistically significant different degree of improvement in terms of sleep (p < 0.001) and medication decrease (p = 0.01). Functional improvement and pain assessment are difficult to measure objectively and may be influenced to varying degrees by patient background, culture, beliefs, mood, and overall health status [35,36]. The differences noted among our dataset and the previously published datasets may reflect these unmeasured confounders. We must also recognize that the bias generated by industry sponsorship could be reflected in the superior clinical outcomes reported in the comparator studies [37].

Limitations of this study include its retrospective nature and therefore lack of control group and lack of standardized follow-up timepoints. This study was also a single-center review, which makes it more vulnerable to lacking external validity or scientific rigor of multi-center studies [38]. While the Stauss et al. study and SENZA-RCT reported standardized 3-, 6-, and 12-month follow-up intervals, only last follow-up data were available for UNMH patients. However, the mean UNMH follow-up time was approximately 18 months, which exceeds the follow-up period utilized by both previous studies. There may exist differences at 3-, 6-, and 12-months among UNMH patients that we are not aware of, due to lack of follow-up data.

This study utilized a patient database maintained by the corporate entity whose product was used to treat these patients, which is a potential source of industry bias. We are reassured that these data are reliable for two reasons. The first is that audio recordings from each patient interview are available from the corporation upon request to verify the outcome reported in the database and the method of its collection. The second is that data from UNMH EMR was used to corroborate 50% of the data reported from the Nevro patient database and was found to agree. That is, there did not seem to be a discrepancy between patient reported outcomes reported to (or from) the Nevro patient database and those reported to UNMH clinicians.

Furthermore, this study, like the SENZA-RCT and Stauss et al. study, measures pain medication reduction through subjective patient-self reports on medication usage. Further studies may benefit from using standardized measures of medication reduction such as MME reduction as a study outcome.

Finally, we compare outcomes and proportions across multiple study designs in the Stauss et al. study, SENZA-RCT, and the present study. The differences in study design may contribute to some differences in data observed between the studies. Finally, assumptions were required to derive data from published sources for the comparison in Section 3.3.

## 5 Conclusion

This non-industry sponsored investigation documented patient outcomes following implantation of HF-SCS for chronic leg and back pain. Our results demonstrate a >70% long-term efficacy for HF-SCS treatment. This is consistent with data reported from the two most comprehensive HF-SCS studies to date. However, improvements in quality-of-life measures and reduction in medication usage were not as robust as reported in industry-sponsored studies. These data complement previously published data and may reflect patient outcomes seen outside of industry-sponsored studies.

## Funding sources

None.

## IRB approval status

Reviewed and approved by the Human Research Review Committee at the University of New Mexico Health Sciences Center; approval #22-162.

## Declaration of competing interest

The authors declare that they have no known competing financial interests or personal relationships that could have appeared to influence the work reported in this paper.

## References

[1] GBD 2016 (2017). Disease and Injury Incidence and Prevalence Collaborators. Global, regional, and national incidence, prevalence, and years lived with disability for 328 diseases and injuries for 195 countries, 1990-2016: a systematic analysis for the Global Burden of Disease Study 2016. Lancet Lond Engl.

[2] Stewart W.F., Ricci J.A., Chee E., Morganstein D., Lipton R. (2003). Lost productive time and cost due to common pain conditions in the US workforce. JAMA.

[3] Dahlhamer J., Lucas J., Zelaya C. (2018). Prevalence of chronic pain and high-impact chronic pain among adults - United States, 2016. MMWR Morb Mortal Wkly Rep.

[4] Urits I., Burshtein A., Sharma M. (2019). Low back pain, a comprehensive review: pathophysiology, diagnosis, and treatment. Curr Pain Headache Rep.

[5] Hoy D., Bain C., Williams G. (2012). A systematic review of the global prevalence of low back pain. Arthritis Rheum.

[6] Krames E. (2002). Implantable devices for pain control: spinal cord stimulation and intrathecal therapies. Best Pract Res Clin Anaesthesiol.

[7] Gatchel R.J., McGeary D.D., McGeary C.A., Lippe B. (2014). Interdisciplinary chronic pain management: past, present, and future. Am Psychol.

[8] Cohen S.P., Vase L., Hooten W.M. (2021). Chronic pain: an update on burden, best practices, and new advances. Lancet Lond Engl.

[9] Patel N., Calodney A., Kapural L. (2021). High-frequency spinal cord stimulation at 10 kHz for the treatment of nonsurgical refractory back pain: design of a pragmatic, multicenter, randomized controlled trial. Pain Pract Off J World Inst Pain.

[10] Orhurhu V.J., Chu R., Gill J. (2022). StatPearls.

[11] Devulder J., De Laat M., Van Bastelaere M., Rolly G. (1997). Spinal cord stimulation: a valuable treatment for chronic failed back surgery patients. J Pain Symptom Manag.

[12] Heidecke V., Rainov N.G., Burkert W. (2000). Hardware failures in spinal cord stimulation for failed back surgery syndrome. Neuromodulation J Int Neuromodulation Soc.

[13] Kumar K., Taylor R.S., Jacques L. (2007). Spinal cord stimulation versus conventional medical management for neuropathic pain: a multicentre randomised controlled trial in patients with failed back surgery syndrome. Pain.

[14] Petersen E.A., Schatman M.E., Sayed D., Deer T. (2021). Persistent spinal pain syndrome: new terminology for a new era. J Pain Res.

[15] Yampolsky C., Hem S., Bendersky D. (2012). Dorsal column stimulator applications. Surg Neurol Int.

[16] Health C for D and R. Senza Spinal Cord Stimulation (SCS) System – P130022/S042. FDA. Published online February 15, 2022. Accessed August 17, 2022. https://www.fda.gov/medical-devices/recently-approved-devices/senza-spinal-cord-stimulation-scs-system-p130022s042.

[17] Shealy C.N., Mortimer J.T., Reswick J.B. (1967). Electrical inhibition of pain by stimulation of the dorsal columns: preliminary clinical report. Anesth Analg.

[18] Verrills P., Sinclair C., Barnard A. (2016). A review of spinal cord stimulation systems for chronic pain. J Pain Res.

[19] Conger A., Sperry B.P., Cheney C.W. (2020). The effectiveness of spinal cord stimulation for the treatment of axial low back pain: a systematic review with narrative synthesis. Pain Med Malden Mass.

[20] Geurts J.W., Smits H., Kemler M.A., Brunner F., Kessels A.G.H., van Kleef M. (2013). Spinal cord stimulation for complex regional pain syndrome type I: a prospective cohort study with long-term follow-up. Neuromodulation J Int Neuromodulation Soc.

[21] Odonkor C.A., Orman S., Orhurhu V., Stone M.E., Ahmed S. (2019). Spinal cord stimulation vs conventional therapies for the treatment of chronic low back and leg pain: a systematic review of health care resource utilization and outcomes in the last decade. Pain Med.

[22] Provenzano D.A., Amirdelfan K., Kapural L., Sitzman B.T. (2017). Evidence gaps in the use of spinal cord stimulation for treating chronic spine conditions. Spine.

[23] Sdrulla A.D., Guan Y., Raja S.N. (2018). Spinal cord stimulation: clinical efficacy and potential mechanisms. Pain Pract Off J World Inst Pain.

[24] Kapural L., Yu C., Doust M.W. (2015). Novel 10-kHz high-frequency therapy (HF10 therapy) is superior to traditional low-frequency spinal cord stimulation for the treatment of chronic back and leg pain: the SENZA-RCT randomized controlled trial. Anesthesiology.

[25] Linderoth B., Foreman R.D. (2017). Conventional and novel spinal stimulation algorithms: hypothetical mechanisms of action and comments on outcomes. Neuromodulation J Int Neuromodulation Soc.

[26] Van Buyten J.P., Al-Kaisy A., Smet I., Palmisani S., Smith T. (2013). High-frequency spinal cord stimulation for the treatment of chronic back pain patients: results of a prospective multicenter European clinical study. Neuromodulation J Int Neuromodulation Soc.

[27] Kapural L., Jameson J., Johnson C. (2022). Treatment of nonsurgical refractory back pain with high-frequency spinal cord stimulation at 10 kHz: 12-month results of a pragmatic, multicenter, randomized controlled trial. J Neurosurg Spine.

[28] Kapural L., Yu C., Doust M.W. (2015). Novel 10-kHz high-frequency therapy (HF10 therapy) is superior to traditional low-frequency spinal cord stimulation for the treatment of chronic back and leg pain: the SENZA-RCT randomized controlled trial. Anesthesiology.

[29] Stauss T., El Majdoub F., Sayed D. (2019). A multicenter real-world review of 10 kHz SCS outcomes for treatment of chronic trunk and/or limb pain. Ann Clin Transl Neurol.

[30] Montaner J.S., O'Shaughnessy M.V., Schechter M.T. (2001). Industry-sponsored clinical research: a double-edged sword. Lancet Lond Engl.

[31] Gazendam A.M., Slawaska-Eng D., Nucci N., Bhatt O., Ghert M. (2022). The impact of industry funding on randomized controlled trials of biologic therapies. Medicines.

[32] Deer T.R., Russo M.A., Grider J.S. (2022). The neurostimulation appropriateness Consensus committee (NACC): recommendations for surgical technique for spinal cord stimulation. Neuromodulation J Int Neuromodulation Soc.

[33] Hagedorn J.M., Deer T.R., Canzanello N.C. (2021). Differences in calculated percentage improvement versus patient-reported percentage improvement in pain scores: a review of spinal cord stimulation trials. Reg Anesth Pain Med.

[34] Cushman D., McCormick Z., Casey E., Plastaras C.T. (2015). Discrepancies in describing pain: is there agreement between numeric rating scale scores and pain reduction percentage reported by patients with musculoskeletal pain after corticosteroid injection?. Pain Med Malden Mass.

[35] Orhan C., Van Looveren E., Cagnie B., Mukhtar N.B., Lenoir D., Meeus M. (2018). Are pain beliefs, cognitions, and behaviors influenced by race, ethnicity, and culture in patients with chronic musculoskeletal pain: a systematic review. Pain Physician.

[36] Krystal A.D., Edinger J.D. (2008). Measuring sleep quality. Sleep Med.

[37] Flacco M.E., Manzoli L., Boccia S. (2015). Head-to-head randomized trials are mostly industry sponsored and almost always favor the industry sponsor. J Clin Epidemiol.

[38] Bellomo R., Warrillow S.J., Reade M.C. (2009). Why we should be wary of single-center trials. Crit Care Med.

